# Foreign

**Published:** 1841-06-19

**Authors:** 


### FOREIGN.

In the 16th number of the present volume, several cases of thymic asthma are copied from the N. Y. Journal. To these we now add the following, from the London Lancet.

*An Example of Convulsive Affection, pervading almost a whole family, with Enlarged Thymus in one of the patients, detailed by the father.*—The father and mother of the children forming the subject of these remarks, reside in Chester-place, Kennington-cross, where their first child (a boy) was born, on the 1st of June, 1826. From his birth he was subject to an unusually relaxed state of bowels; at nine months old, before he had cut any of his teeth, he was threatened with a fit; his nervous system experienced an extraordinary degree of excitement; his eyes became very bright, and very much protruded; his cheeks very much flushed, and his thumbs were drawn in towards the palms of his hands. These symptoms continued for several days, but they passed away without any fit taking place; the relaxed state of the bowels continued, and the child gradually became very much emaciated.

Dr. Birkbeck was consulted, who thought the state of the stomach proceeded from improper diet, or from over-feeding, and treated the case accordingly, prescribing a very exact regimen. The child did not improve under this treatment, and was placed under the care of Dr. Walshman, in the neighbourhood, who thought his complaint lay in the head. He administered calomel very freely; for several weeks the child took four grains a day constantly; a great deal of chalk, also, was administered. Under this treatment he gradually recovered, and, although he continued a weak and nervous child, his general health became good. When about two years old, his mother observed a great peculiarity in his manner of breathing, particularly when awaking from sleep; he emitted a sharp, whistling kind of noise, very like what she has since found to be occasioned by croup; but no other symptoms of croup, or of any other complaint, followed. The boy is now nearly fifteen years old; he has had several attacks of determination of blood to the head, (*a*) which have, at different times, made it necessary to withdraw him from his studies, but he now enjoys good health.

The next child was a girl, born also at Chester-place, in December, 1827. This was a healthy child from her birth, until she attained six years of age, when she became very subject to croup; the attacks, however, were always dealt with in time, and have gradually become less frequent. During the last few years, she has been subject to severe bilious attacks. (*b*)

Before the birth of the next child the family removed to Southville, in the Wandsworth-road, where all the succeeding children were born, and the family have had the advantage of the medical attendance of Mr. Munpress, of the Wandsworth-road. A girl was born in July, 1829; this child continued healthy until she was about two years and a half old, when she experienced a kind of fit; falling back suddenly, without any previous symptoms, her respiration became suspended, her eyes fixed, and her limbs stiffened, but *no* convulsions took place. On being removed into the open air she recovered; and though apparently a little distressed for a short time afterwards, no other consequences ensued. On two other occasions within the following six months, similar fits occurred, but they have never since returned; and, although she continues a delicate looking child, her general health has ever since been good. (*c*)

The next child was a boy, born in June, 1831. This was a very fine child, and continued perfectly healthy until he was nine months old, when he began to make a crowing, whistling noise, whenever he was at all excited, and frequently when he awoke from sleep; these crowing inspirations became louder and louder, and, following each other five or six in succession, produced a kind of spasm, from which he had some difficulty in recovering his breath; and on several occasions, when that difficulty became greater than usual, he went off into fits, exactly similar to those described as occurring to the last child. Dr. Walshman's aid was called in, in this case; he thought the child had all the symptoms of hooping-cough, except the cough. He acknowledged that he was at a loss how to treat the case, and attempted but little in the way of medicine. Leeches were occasionally applied, both to the head and the chest; and an ointment, of which belladonna was the chief ingredient, was used to the throat, which appeared to be the seat of the complaint; calomel was administered internally, and occasionally assafœtida. The general health of the child did not suffer from the symptoms described, nor did he lose flesh. The whistling inspirations, the spasms, and occasional fits, continued until he was about two years old; (*d*) they then ceased, but he was for some years afterwards subject to occasional attacks, in the early part of the night, after his first sleep, when he would awake up in apparently an unconscious state, trembling violently, and screaming, as if in pain. These symptoms were always allayed by putting the feet in hot water, and making cold applications to the head. These last attacks have gradually disappeared; the child, now about ten years old, is of a nervous temperament, and subject to determination of blood to the head, and it is on that account found necessary to check his natural inclination for study.

The next child was another boy, born in October, 1832. He was quite well till about four or five months old, when the same crowing inspirations came on, followed by similar spasms and fits as in the last case. The same treatment was adopted; calomel was administered pretty freely; leeches were repeatedly applied, both after the recurrence of fits, and whenever the frequency of the spasms made it likely one would occur. The belladonna ointment was also used again constantly; a cough supervened in this case, by which the child was greatly distressed, and he became very much reduced. In this state he was taken to Sir Charles Clarke, but, it is feared, at too late a period to be of service; the poor little fellow got worse and worse, and died a few days afterwards, whilst apparently struggling with one of the spasms which have been described. On a post mortem examination of this child, the lungs were found much inflamed, and full of tubercles; to which causes the death was attributed, and attention was not at that time directed to the thymus gland.

At the time of this child's death, the mother was again pregnant, and she herself entertained a great dread that the expected child would be similarly affected with the others. It was born in February, 1834, and proved to be another boy; but he was free from any symptoms of the dreaded complaint until he was about two years old: he then began to emit the crowing inspiration whenever he drank, but at no other time; and even this did not continue long, but he became subject to glandular swellings in different parts of the body; (*e*) the stomach became very much enlarged; and, on receiving a slight blow from an orange on the upper part of the arm, a swelling took place there, which it became necessary to open, when about a wine-glassful of pus was discharged from it. This child has had no further appearances of the family complaint, but has always been very subject to croup, and has still a tendency to it, as well as to swellings in different parts, particularly about the lips.

The next child, another boy, was born in October, 1835; before he was three months old the crowing inspiration began, and was quickly followed by the spasms and fits. (*f*) The same treatment as before was observed, and continued for several months. In June, 1836, the spasms had become very frequent, occurring sometimes twenty times a-day, when the parents determined to try the effect of change of air, and they took the child to Margate; and, extraordinary to say, they had no sooner set foot on board the steamer, than the spasms and crowing ceased altogether! But although the complaint was thus for a time got rid of, the child, during the fortnight he remained at Margate, became extremely debilitated, and fell away to a mere skeleton, although he had not previously lost flesh during the continuance of the spasms and fits. At the end of the fortnight he was brought home, and by dint of nourishing diet and great care, he soon recovered his flesh, and continued in excellent health for nearly a year; at the end of that time, the crowing noise, the spasms, and the fits, all returned. As soon as this happened, he was taken to Gravesend; the effect of the change was not so instantaneous as before, but after a few days (during which he had a frightful fit) the complaint again disappeared. (*g*) The child did not lose flesh as at Margate, but was brought home in good health, and continued so for another year. At the end of that time, without any previous return of the usual crowing noise, or of the spasm, he was seized with a severe fit in the middle of the night, which was fortunately noticed by the nurse, and he was with great difficulty recovered from it. Another year of health was granted to him, during which he became a very fine child, and nearly reached his fourth year; his parents then took him to Herne Bay for a fortnight. He returned apparently in perfect health; but a few days afterwards, without any of the former symptoms, he was suddenly seized with strong convulsions, and after struggling with them for five or six hours, died on the 2d August, 1839. (*h*)

The next child was a girl, born in April, 1837; before she was a month old the mother's ear detected the crowing, or, rather, in this case, the whistling inspiration. It was not, however, so strongly developed as with the boys: when about two months old she had a slight fit, (not a convulsion,) from which she soon recovered, and has never since had any symptoms of the complaint; when a little more than three years old, she had rather a severe convulsive fit, but this is supposed to have been caused by indigestion. She is now in good health, but is a very excitable child. (*i*)

Another boy was born on the 1st of February, 1840; the mother was pregnant with this child when the last boy died. At three months old the crowing inspiration began to be slightly perceptible, so slightly that it was hoped it would disappear again, as in the last case of the girl. It continued, however, occasionally, but still slightly, for several months, and then the spasms and fits came on as with the other boys. The child's general health continued good; but when excited by the sight of its food, or by any other cause, and always when awaking from sleep, the crowing noise was produced; the eyes protruded, the thumbs were drawn in, and if the spasm was not quickly relieved, the child would fall back insensible; the eyes fixed, the tongue thrust out and nearly black, the breathing suspended, and the limbs stiffened; until, by throwing water in his face, by putting salt in his mouth, or by carrying him into the open air, a reaction had been produced, and with a gasp he recovered his breath.

The former remedies, calomel and leeches, were again had recourse to; iodine was substituted for belladonna, and assafœtida was again administered. No improvement, however, took place; and remembering the effect of removal in a former instance, the child was taken to Gravesend, early in December last. The change was evidently of service, and after an absence of a week, he was brought home something better. The crowing noise and the spasms had not ceased; but he had no fit during the time he was absent. He had a slight fit on the day he returned home, probably from the excitement of seeing again his brothers and sisters. He continued free from fits during the severe frost which followed, and which lasted until the middle of January; but as soon as the thaw came on the complaint returned, stronger than ever; and before arrangements could be made for again changing the air, two very severe fits followed each other on succeeding days, leaving the child for a few hours after each fit in a very distressed state. During the last of these attacks the child became perfectly blue: for one day he was spared a fit, but the next day—with every appearance of health about him—whilst lying in his cot, smiling at the endearments of his little sister, he suddenly put up his finger, as he had been accustomed to do, as if to call attention to the striking of a bell in the neighbourhood, and in the next moment fell back in a fit, from which, unhappily, he never re-recovered. Every means that those about him could think of were resorted to, to restore animation; but before medical aid could be procured, the child was quite dead. A post-mortem examination has been made in this instance, (*k*,) and the thymus gland has been found inordinately enlarged; being nearly four inches in length, two and a half in breadth, and more than half an inch in thickness; and had, of course, been subject to still greater enlargement, when filled with blood by any unusual excitement. The gland was found to cover and press upon a part of the windpipe; thus accounting for the difficulty in respiration. It pressed also upon the veins of the throat: thus, in moments of excitement, preventing the free return of blood from the head, and so accounting for all the symptoms which have been observed, not only in this child, but in all the other cases above described. The heart and lungs were found perfectly healthy; showing that the complaint was entirely a local one, or, at any rate, proceeded from a local cause, and not from any general disarrangement of the system.

Such being the history of this case as affecting this family of children, the following inferences may be drawn from these observations: viz., that the complaint does not necessarily affect the general health of those who suffer from it, although it seems to have a tendency to produce a fulness of blood to the head; that girls are less subject to the complaint than boys; and that change of air, and particularly that sea-air has a decidedly favourable effect on it. The great point, however, to arrive at, is what course of medical treatment should be pursued in attempting to grapple with the original cause of the complaint; viz., the unnatural enlargement of the thymus gland.

*Remarks by the medical attendant of the family.*

(*a*) The affection of the head, in this case, on the occasions that I have been consulted, appeared to depend on gastric irritation, and has generally yielded to stomachic remedies.

(*b*) Sick headachs are the prevailing ailments of this case, which are usually removed by brisk cathartics.

(*c*) Since infancy no disturbance of the cerebral or respiratory organs have been observed. She lately had an attack of dyspepsia, occasioning an irritable state of skin, producing erythema.

(*d*) The attacks in this instance were so violent, inducing such a constriction of the rima glottidis as to produce asphyxia, and almost suspended animation. He has lately been indisposed with plethora of the cerebral mass, which yielded to an abstinent regimen, and aloetic saline cathartics.

(*e*) This child is of a strumons habit of body, and, when indisposed, derives benefit from a treatment appropriate for such a constitution.

(*f*) The spasms alluded to were produced by the closing of the aperture into the larynx, producing the peculiar sonorous respiration.

(*g*) The effect attributed to change of air I always looked upon as inexplicable, perhaps a mere coincidence.

(*h*) Depletion, purgative, and fœtid enemata, cold to the head. Sinapisms and warm bath were persevered in during the whole period of the convulsion without effect.

(*i*) The child's health is generally tolerably good.

(*k*) Having little doubt that this was a decided case of laryngismus stridulus, it became an object after death to see the state of the thymus gland; the opportunity being afforded, its state was found to be as described, and readily accounted for the symptoms detailed; all the other viscera in the chest and abdomen appeared to be in a normal state: the head was not opened.

---

*Case of Inflammation of the Absorbents, and Erysipelas from Chilblain.*—S. M., aged 23, was admitted January 6, 1841, under the care of Mr. Liston. She is of slight stature, and of the nervous temperament; she appears to be labouring under inflammation of the absorbents in the left leg and thigh, resulting from the irritation of a chilblain on the great toe of the same side. Numerous bright red streaks are seen running from the dorsum of the foot up the front and inner side of the leg, rather behind the prominence of the internal condyle of the femur, and up the inner side of the thigh to the groin; there is some hardness along this course, and great tenderness on pressure; there is no general enlargement of the limb perceptible; the streaks are very distinctly marked, being hardly broader than one-eighth of an inch, and, occasionally, running into one another. The patient complains of a feeling of soreness and burning in the inflamed parts; she has violent headach, and is very restless and uneasy; her bowels are rather costive; her tongue thickly coated, and brown at the back; her mouth is "much out of taste," and she complains of thirst and dryness of the fauces; the pulse is quick and irritable, but not full. She says that the chilblain on her toe has been troublesome for some weeks, that three days ago she walked about four miles, an unusual distance for her, and that the next day the pain up the leg commenced. There is no enlargement of the glands in the groin, although there is some pain and tenderness in that region. The patient to be kept in bed with the affected limb raised on an inclined plane and pillows as high as she can conveniently; the limb to be fomented every two hours with flannels wrung out of hot water, which are to be changed every two or three minutes foi about half an hour; water-dressing to chilblain; to have eight grains of extract of colocynth, and four of calomel at bedtime, and a black draught in the morning. Middle diet.

7. The medicine did not operate freely this morning; the house-medicine was, therefore, repeated with good effect; there is rather less tenderness and redness than yesterday, and the redness is of a duller cast; tongue still very much furred. To have a draught, containing two drachms of Epsom salts, five grains of carbonate of magnesia, one-eighth of a grain of tartarised antimony, and an ounce and a half of water, every four hours. Continue the fomentations.

11. Yesterday the thigh and leg looked considerably better, but to-day the redness has become a little brighter, and more diffused; over the foot and lower half of the leg it has much the appearance of erysipelas; the red parts are elevated above the healthy skin, and feel very hot; the streaks of the thigh are almost invisible. Mr. Liston made a number of punctures with a lancet over the inner and front part of the leg where the redness was deepest; the limb was then allowed to hang over the bed, and was fomented until it had ceased bleeding; several ounces of blood flowed, and the patient expressed herself much relieved afterwards.

13. Punctures repeated on the other side of the limb, the inflammation having extended to that side since yesterday morning. The thigh is now quite natural, the redness not extending higher than the upper edge of the patella. The patient is now less feverish; pulse is quieter, about 98; bowels open; tongue, however, is still foul, and breath rather offensive. To take seven grains of mercury with chalk every other night, and the draught twice a-day.

15. Much improved; redness fading every where; there is a part over the external condyle which is very œdematous, and has a soft boggy feel; there is, however, no feeling of fluctuation; there are several large bullæ on the outer side of the foot, and over the heel.

17. Inclined plane removed; leg supported only on pillows. Discontinue the medicine. To have a tablespoonful of castor-oil every third morning, if her bowels are not opened regularly. Full diet.

23. The redness has now entirely left the leg and foot, the skin of which looks shrivelled and dry. Patient walks about a little, but says that the leg feels very stiff, and swells a good deal in the evening. To have a bandage applied from the toes to the knee, and to keep the limb rested on a chair.

27. Quite well, cuticle desquamating; general health perfectly good. Discharged, cured.

*London Lancet.*

---

Treatment of Pruritus Ani.—*To the Editor of the Lancet.*—"A Subscriber" relates, in the Lancet of the 23d ult., an intractable case of pruritus ani, and wishes to be informed "how to cure the above most troublesome affection." Having been an occasional sufferer for some years, I have much pleasure in stating what means have afforded me the greatest relief.

I believe that the affection is not essentially a local disease, but arises from a state of system in which small hard portions of feculent matter lodge in the folds of the mucous membrane of the lower bowels, producing a morbid sensibility of the extremities of the nerves about the anus, in the same manner that the irritation of a stone in the bladder is referred to the glans penis; although, by a long continuance of the complaint, and by the constant manifestations which the unfortunate patient is constrained to practise, keeping up the irritation, the morbid sensibility of the nerves of the part is rendered permanent, and the disease then assumes a local character.

During a recent attack I took, at bed time, two grains of mercury with chalk, with three of Dover's powder, and on the following morning—

Castor oil, ʒiij;

Liquor potassæ, ʒss;

Cinnamon water, ℥j.

This plan was persevered in for a fortnight, when the gums became rather tender, and all irritation subsided, while the bowels acted naturally, and the secretions were perfect. Since that period I have been but little troubled with the complaint. The occasional injection of warm water into the rectum, and sponging with the same at bed time, are certainly very useful, and should not be neglected. I have also used, with advantage, five grains of the soap and opium pill as a suppository at night, followed by a mild purgative in the morning. Violent exercise is hurtful. I have found ripe fruit and vegetables very beneficial.

Hoping that the above remarks may be deemed of sufficient interest to obtain a place in your valuable publication, I am, sir, your obedient servant,

A General Practitioner.

London, Feb. 10, 1841.

P. S.—Kreosote ointment, in the proportion of one drachm to an ounce of simple cerate, and the solid nitrate of silver, applied to the verge of the rectum, have, in some cases, been successful. I have tried the former in my own case, but without any benefit.

—

Another correspondent, Mr. Williamson, advises the use of cold water sponged over the part several times in the day; and the zinc ointment, diluted with one-half of simple cerate, to be applied after each ablution. To the local treatment may be added, with advantage, the exhibition of the following pills, one of which is to be taken occasionally:—

℞.—Extract of colocynth;
Extract of hyoscyamus:
Blue pill, of each, ʒj.

Make into twelve pills.

Mr. Williamson also enjoins abstinence from stimulating aliments and vegetables. He observes, "leeches are by no means to be employed."

—

*Treatment of Pruritus Ani.—To the Editor of the Lancet.*—A correspondent inquires, through your excellent periodical, the best method of curing pruritus ani. It has already been replied to, but, I am fearful, in part unsatisfactorily; nor do I see how it could be otherwise, as it will be found to proceed from various causes, locally and constitutionally. In proof of which, on referring to my notes on the subject, I find the three first cases of simple pruritus recorded are totally different.

In the first, the cause of the pruritus proceeded from two slight ulcers. Ointments and lotions had been recommended; no examination being previously made; satisfied by knowing there was an "itching." Here, I believe, the solid nitrate of silver, from experience, is the very best remedy; applied, by simply touching the ulcers with the blunt extremity of the caustic, every alternate morning. It occasionally causes some pain; but it is quickly followed by a coolness of the part, and a great diminution, if not a total subsidence, of the itching for that day; and, finally, effects a complete cure. The length of time depends much on the habits and constitution of the patient.

In the second, the pruritus arose from a very slight excrescence: I have found two or more existing. These I first snip off, and then touch the bases with the butter of antimony, or nitrate of silver; either of which quickly effects a complete cure.

In the third, the pruritus was found to be caused by the lower part of the gut being studded with small white elevations—pimples. Either of the three following applications will rapidly bring about a cure; first, a strong solution of acetic acid in water; or secondly, a strong solution of the bi-chloride of mercury; or, thirdly, a solution of sub-borate of soda. The latter was very strongly recommended by Dr. Dewees; and I have always found it answer well where the patients reside at a distance, and I have been prevented from knowing the condition of the parts; I have, on many occasions, ordered them to foment, and directly after to introduce the ointment of nitrate of mercury diluted, the size of a nut every night; and take, the following morning, a good dose of Ward's paste, carefully prepared after Gray's formula, with the best result.

Old women, in the country, order the use of "rope-yarn" untwisted: which would suggest to us tar ointment; but as I do not remember ever being baffled in a single case by the remedies I have related, I have not had occasion to try its merits. But, in conclusion, I must strongly recommend that any irregularities of the constitution be righted, and also warm fomentations frequently used, or our remedies will avail us but little.

For the present, I must leave the subject to others, who may be willing to devote a little of their spare time to the subject, and perhaps will enter more deeply into the subject on a future occasion. I have the honour to be, sir, your obedient servant.

J. H. Horne, Surgeon, &c.

—

*To the Editor of the Lancet.*

Sir:—I am somewhat surprised at so much being said, in the pages of the Lancet, about that simple, though troublesome complaint, "pruritus ani." I have been a severe sufferer from it myself; but neither in my own case, nor in many others, have I found any difficulty in curing it. Of course, eczema, and the irritation arising from the presence of ascarides, require each a different treatment; but the itching which induces one to scratch till the part is raw, and the pain intolerable during sleep, I have always found to yield quite readily to three or four doses of the ordinary lenitive electuary; and the application, night and morning, of a cooling ointment composed of—

Spermaceti ointment, ℥ss;
Prepared chalk, ʒss;
Liquor of the subacetate of lead, ℥ xx; M.

From two to eight days generally complete the cure. I am, Sir, yours obediently,

Henry Barham Harris, M. D.,
Physician to the Dumfries and
Maxwell-town Dispensary.

Dumfries, Feb. 27, 1841.

—

A correspondent, H. P. S., has found new oakum, or any preparation of tar, a valuable remedy in this affection; perhaps a lotion of creosote, or creosote ointment, might be found serviceable.

—

*To the Editor of the Lancet.*

Sir:—Your correspondent, J. H., solicits the suggestion of a remedy for the above very troublesome complaint. Although very common among children, it is not so among adults; but, for the last half century, I have been a painful witness that it is sometimes, and I believe generally, in adults, incurable.

I have tried every remedy from turpentine enemata to frequent aloetic purgatives; and I believe that, in most cases, temporary relief may be had by occasional purging with extract of colocynth, continued for four or five days successively; and, at the same time, taking every morning a small dose of sulphate of magnesia; and daily injecting, far up the bowels, a solution of sulphate of iron in decoction of aloes. This treatment will bring away many worms, and will insure comfort for two or three months, or perhaps longer; but I fear the complaint does not admit of radical cure; and, trifling as it appears, I must acknowledge that it has added not a little to the discomfort of a life now hastening to a close. I am, Sir, respectfully,

AN OLD PHYSICIAN.

—

*To the Editor of the Lancet.*

SIR:—A correspondent, in a late number of your valuable Hebdomadal, who signs himself J. H., states that he has a patient suffering from "pruritus ani," occasioned by ascarides, and desires to know an effective mode of treatment for removing them; I, therefore, beg to direct his attention to a very certain and innocent remedy; viz., the dolichos pruriens of Linnæus; which was, I believe, introduced to the notice of the profession by the late Mr. Chamberlaine, of Clerkenwell, who published a practical treatise on the superior efficacy and safety of cowhage in diseases occasioned by worms; and having frequently had recourse to this remedy in the form of an electuary in my own practice, I am able to bear testimony of its success in completely exterminating those annoying and debilitating vermin from the mucous membrane of the intestines.

The following is the form in which I have been accustomed to administer it—

℞ Dolichos pruriens, ʒij;

Treacle, ℥iv; M.

A tea-spoon, dessert-spoon, or a table-spoonful to be taken night and morning (according to the age of the patient) upon an empty stomach. It is necessary to continue the remedy for ten days or a fortnight; during which period a dose of scammony and calomel should be administered twice or three times, to remove the dead worms which may cling to the rectum. I am, Sir, your obedient servant,

EDWARD DIXON, Surgeon, &c.

---

*On the Congenital Opacity of the Cornea.* By JOHN CHRISTIE, M. R. C. S. L.—While on a recent visit to Aberdeenshire, I was requested by Mr. Proctor, surgeon, Towie, to visit, along with him, a child of three months of age who had been born blind. On examining the eyes the corneæ were found to be of a pearly or blueish-white colour, deeper and more opaque at their centres than their circumferences. The irides were indistinctly seen through them, and, although the pupils were dilated and irregular in form, it was evident from their sluggish motions that they were sensible to the stimulus of light. Both eyes appeared to be small, flat, and *undeveloped*, and were in a continued state of oscillation. Mr. Proctor and the mother of the child said they were then considerably clearer than they were at birth. The eyes and their appendages were perfectly healthy, and stated to have been so since birth, at which time the opacity was first discovered by the nurse.

Mr. S. Crompton, in the Medical Gazette of the 12th of December last, records several instances of congenital opacity of the cornea that have occurred both in his own practice and in that of others, particularly in that of Mr. S. Farar, of Deptford; to the two first of which that above related agrees in almost all particulars.

Mr. Middlemore, of Birmingham, it would appear, doubts the occurrence of congenital opacity of the cornea, but the cases now on record, although few in number, leave no question as to the possibility of such an event, and the veracity of Messrs. Crompton, Walker, and Barton*, cannot be called in question, however it may run counter to the preconceived notions of Mr. Middlemore, high as is his authority in ophthalmic surgery. Assuming, then, that congenital opacity of the cornea may, and has more than once existed beyond the possibility of doubt, it appears to me that its occurrence may be explained by three causes, all different in their nature and effects. In the two cases recorded by Mr. Crompton, as occurring under his own eye, Messrs. Allen and Barton agree with him in regarding the eye as in an *undeveloped* state, and in the instance which I have related it is one of the most obvious conditions. Mr. Farar, however, does not say whether in the instances that fell under his observation the eyes were in this state; he merely notices the opacity of the cornea; but from their similarity to those already spoken of, so far as can be learned from his paper as quoted by Mr. Crompton, it is not unfair to infer that they were of the same nature; the opacity originating in the same cause—arrested development. The fœtus, we know, is subject to many diseases in utero, and arrest of development of one or other of its organs is a matter of every day observation. On what grounds, then, can the eye be exempted from the same laws as frequently influence the other organs of the body? and if this is granted, why deny its influence, or reject it when obvious as a sufficient cause of congenital opacity of the cornea?

Mr. Crompton also quotes a case from Mr. Walker, of Manchester, in which, on the second or third day after birth, the corneæ were found to be "opaque throughout, and unusually large and prominent, so that very little of the sclerotic was discernible. The opacity was of a bluish white colour: there was scarcely any irritation about either eye: nothing like inflammation," but in the second year the corneæ were "perfectly transparent and of normal size." It is at once obvious in this case, although there were opaque corneæ, that the condition of the eye was different from that in the cases already commented on. And, if together with the ap-

* Vide Mr. Crompton's paper in Med. Gaz., Dec. 12, 1840.

pearance of the eyes, we take the satisfactory progress of the case, so that in two years complete vision was restored, we are led to conclude that the opacity resulted from a redundancy of the aqueous humour, not from congenital arrestment of the development of the eyes.

The due amount of the aqueous humour evidently depends on a happy balance being struck between the functions of its secreting and absorbing organs; hence the *rationale* of the production of the opacity in this instance is easily understood. The function of absorption of this humour being impaired, whilst that of its secretion continued normal, opacity of the cornea, from over distension of the anterior chamber, was the result.

Again, in another quotation from Mr. Walker given by Mr. Crompton, a case is detailed where there is every reason to believe that purulent ophthalmia, ending in destruction of one eye, and great impairment of vision in the other, had run its course in utero. This, although hitherto an unnoticed occurrence, cannot be denied, resting as it does on so respectable evidence as that of Messrs Crompton and Walker. Intra-uterine pathology is but in its infancy, yet the researches of Graetzer, Simpson, and others, have revealed enough to make us cease to wonder, as year after year witnesses new contributions to this department of pathology, which overturn and root out opinions and dogmas that have "grown with our growth and strengthened with our mind."

From what has been said I would infer, as already stated, that congenital opacity of the cornea may occur in three ways:

1st. From arrested development of the eye.

2d. From loss of balance between the functions of secretion and absorption of the aqueous humour, occasioning over-distension of the anterior chamber of the eye, and consequent opacity of the cornea.

3d. From intra-uterine purulent inflammation of the eye.—*London Med. Gaz.*

---

### *On the Operation for Talipes.*

*To the Editor of the Medical Gazette*—In the commuication you did me the favor to insert in your number of the 11th of December last, I promised you a report of some cases, illustrative of a new operation for the cure of a certain variety of talipes, for which the Stromeyerian operation was inapplicable. I shall now endeavor to fulfil my promise, although my avocations compel me to do so very briefly. Besides its reference to those cases of talipes, it is moreover interesting as calculated to meliorate many other infirmities arising from total paralysis, or diminished power, of certain classes of muscles.

In the month of June, 1840, I had my attention directed to this subject by observing the peculiar gait of a gentleman who was passing along the street. Although I neither knew nor spoke to the individual, I was struck with the manner in which he walked, and took more notice of it from the circumstance of being at the time much engaged in the practice of the Stromeyerian operation for the cure of club-foot. That operation consists essentially in the division of the tendons of muscles, which act too energetically, and extending them, before the lymph which is thrown out to cement the divided extremities together has become consolidated: so that, in the end, there is a new portion actually implanted between the divided extremities of the originally contracted tendon. That this is not a speculative idea I can prove by specimens in my possession. I observed that in the variety of the malformations referred to—evidently caused by paralysis—there was no morbid tension; but, on the contrary, a morbid relaxation. The idea occurred to me, that if a portion of the tendon were cut out, and the divided extremities made to reunite, by being kept in contact during the healing process, it would at least improve the flapping condition of the weak member, and by retaining it in a state of greater tension, might produce a tendency to contraction in the paralysed muscles. On my return home I explained my views to my intelligent friend and patient, Mr. Rhind, Surgeon, Edinburgh, who was then under my care for the cure of congenital talipes varus, of the worst degree, in both feet. I told him that the propriety of the operation was so strongly impressed upon my mind that I was determined to adopt it the first time a case of the kind referred to came under my care. He expressed himself much pleased, and considered it a fortunate idea. It was not long ere I had an opportunity of testing the value of my proposed new mode of practice, in the case of Miss ——, which I shall now detail.

Case I.—Miss ——, æt. six years, has been deprived of the use of the left leg, by a paralytic stroke. She has undergone a variety of treatment under some of the first medical gentlemen of this town; but, for the last two years, has been given up as a hopeless case. Her present condition is this: the left leg is perfectly powerless, dangling by the side of her crutch, without reaching the ground, and is much colder than is natural; the foot assuming the appearance of a slight degree of varus, so that it would, if brought to the ground, rest on its outer edge, the toes inclined a little inwards, and the heel slightly elevated. On the 30th of June, 1840, assisted by Mr. Rhind, my son, and one of my apprentices, Mr. Pacey, I made a longitudinal incision along the course of the peronæus tertius, which I elevated, and excised a portion of it, to the extent of three-sixteenths of an inch. I then closed the wound with plaster, and applied splints and bandages, so as to approximate the divided ends, and maintain them in contact.

2d day.—Has been free from complaint.

4th.—Removed the plaster, and, after bathing the foot, again applied plaster and bandaging as before. Care has been taken. during the dressing, not to extend the foot so as to separate the recently approximated extremities of the divided tendon.

6th.—All going on well; and now feels the foot and leg quite warm.

7th.—Has entire command of the leg, and so much strength in the foot, that I requested her to take hold of my hand, and try if she could use her limb, when, to the surprise of all present, she was able to walk across the floor of my surgery.

10th.—In the presence of my talented friend, Dr. J. L. Bardsly, and others, she actually walked across the room and back again without any assistance whatever; a fact which equally astonished and delighted him and all present. In twenty days she was enabled to walk about in a laced-up boot; and in a week more to throw her crutches aside entirely. She has continued well and strong ever since. Besides the operation, nothing else was done in this case, excepting the use of a stimulating liniment, which she had employed before without the least benefit.

Case II.—Miss ——, æt. 10, has been suffering from an attack of valgus of the right foot for the last six years; the malleolus internus nearly resting upon the ground when supporting the weight of the body on this leg: not from contraction of any muscles, but from a paralytic state of the tibialis posticus and flexor longus pollicis pedis of the right foot. With the same assistance as in the former case, I excised nearly a quarter of an inch of the tendons of the tibialis posticus and of the flexor longus pollicis pedis, and dressed and bandaged as before, so as to keep the divided extremities in contact. All went on perfectly well, and from the day the operation was performed, the patient said the foot felt warm, although previously the foot and leg were constantly cold. In three weeks she could walk quite straight; the foot and ankle being maintained in their proper relative position.

Case III.—The next case was Mrs. ——, æt. fifty, who had suffered from varus of the right foot from infancy, arising chiefly from a paralytic state of the peronæus and longus brevis. On the 6th of July, 1840, I excised three-eighths of an inch of these tendons, above the malleol. extern., closed the wound with plaster, and bandaged it so as to keep the ends of the divided tendon as much approximated as possible.

2d day.—Quite free from constitutional irritation, and all complaints.

3d.—Makes no complaints; says she feels the foot much warmer, and can now move the toes for the first time in her life. Every thing went on in the most satisfactory manner in this case; and in twenty-one days she was enabled to put on a laced-up boot, provided with a steel support, which enabled her to walk about with much more comfort than formerly. From want of attention the improvement did not continue so manifest as at first. The experience I have now had leads me to believe that this may be partly attributed to the circumstance of the tendo-achillis being slightly contracted. Had I such a case to operate upon now, I would divide and lengthen the tendo-achillis, whilst I would take a portion from, and thus shorten the other tendons referred to.

Case IV.—Master Gresty, æt. 6½ years, has had talipes valgus since one year old, arising from absolute paralysis of gastrocnemii and other muscles on the back of the leg; so that there is not only total inability of extending the foot, but such relation of all the extensor muscles, as to allow the foot to be pressed upwards until it touches the front of the leg. On the 4th of September, I excised three-eighths of an inch of the tendo-achillis.

3d day.—All going on well.

5th.—Makes no complaint; wound nearly closed, and very little matter discharged.

7th.—All going on well, and still great warmth of foot and leg, which has increased since the operation, although there is no pain or inflammation to account for it.

10th.—Wound nearly closed; tendon so firmly united as to resist any attempt at pressing the foot upwards, excepting a little beyond a right angle with the leg; and has now power of flexing and extending the foot freely; showing that the paralytic state of the extensor muscles is completely overcome. At three weeks after the operation, this patient fell and injured the leg, so as to cause inflammation and suppuration around the newly formed cicatrix, which retarded his recovery. After this had subsided, with the use of stimulating embrocations, this patient regained the use of his extremity, so that he could, in three months after the operation, walk with ease; flexing and extending the foot freely, and capable of supporting the foot and leg in its proper relative position. Before the operation, any attempt at walking was attended with great awkwardness; the leg being tossed forward, the toes turned very much outwards, and the inner ankle approximating the ground.

Case V.—Master B——, æt. 12½ years, from a paralytic seizure or accident, they cannot tell which, has been much in the same state as the last patient, for about a year. In October, 1840, I excised five-eighths of an inch of the tendo-achillis, dressed and bandaged as usual.

3d day.—All going on satisfactorily.

4th.—Makes no complaint; wound nearly closed, and very little matter effused; dressed and bandaged as at first.

6th.—Wound almost entirely closed, and has now the power of flexing and extending the foot freely, indicating the reunion of the tendo-achillis, as well as restoration of retractile power. Foot and leg still kept bandaged in the same manner as at first.

8th.—All going on most satisfactorily.

In two months put on a laced-up boot, with which he walked freely; has continued without complaint, and can now walk nearly as well as if he had never been so afflicted.

Case VI.—Master Moss, æt. 10 years, has right leg quite useless from a paralytic seizure in infancy. The leg not only powerless, but also cold and withered, being about half the size of the other one. The knee bent to an angle of forty-five degrees; the foot turned outwards, and can be easily pressed up against the tibia, showing the extreme want of tone of the gastrocnemius and tibialis posticus. I excised three-eighths of an inch of tendo-achillis, and about as much of tibialis posticus; dressed and bandaged as usual. All has gone on well; and he is provided with a high-soled boot, to compensate for the shortness of his leg. He can now walk without a crutch, merely requiring the assistance of a stick. This leg is much grown, and greatly warmer than before the operation; indeed, he is altogether stronger.

Case VII.—Miss ——, æt. 20 years, a case of calcaneo-valgus of left leg, of twelve years standing, arising from total paralysis of gastrocnemius, with contraction of tibialis anticus, extensor longus pollicis pedis, extensor longus digitorum pedis.

On the 25th of September, 1840, I operated, by excising a full one-half of an inch of the tendons of the tendo-achillis, and dividing the tibialis anticus, extensor longus pollicis, extensor communis digitorum pedis. Applied plaster to the wounds, and splints and bandages, so as to maintain the extremities of tendo-achillis in apposition, and those of the other parts.

2d.—All going on well; makes no particular complaint, and free from every symptom of constitutional irritation.

3d.—Still going on well.

4th.—Removed the dressing; wounds in the anterior part of the leg closed; that in the posterior looks well, is nearly closed, and very little pus discharged from it. Dressed, and applied splints and bandages as before.

6th.—All going on well, with the power of flexing and extending the foot; showing the reunion and restored muscular contractility of the tendo-achillis and gastrocnemius muscles, as well as the antagonist tendons on the anterior part of the leg. From this time all went on so well as to require no particular remark; and in six weeks she wore a laced-up boot, walked with comparative ease, and is now comparatively well and strong. As in the other cases, the leg, which was cold and powerless, acquired an increased degree of heat as well as strength. To aid this, and strengthen the limb more rapidly, I recommended friction with stimulating liniment, as in the other cases.

Case VIII.—Wm. Bowker, Sheffield, æt. 7 years, has had talipes valgo-calcaneus from infancy, supposed to have arisen from a paralytic stroke when six months old. Has no power of gastrocnemii, so that the foot can be pressed upwards until it almost reches the tibia. When walking, the foot is turned very much outwards, and the malleolus internus nearly touches the ground. On the 26th of February, 1841, I operated by excising three-sixteenths of an inch of tendo-achillis, and dressed and bandaged in the usual manner for such cases.

2d day.—All going on well.

4th.—Makes no complaint; removed the bandages, &c., and reapplied them as before. There was the smallest possible quantity of pus under the plaster.

6th.—Can now flex and extend the ankle-joint and toes. From this time continued to progress in the most satisfactory manner, and in eighteen days could walk easily without any apparatus applied to the foot or leg, having completely regained the power of the gastrocnemius and tibialis posticus and flexor longus digitorum pedis.

I could easily multiply cases of a similar nature, but shall only add one more, where I excised about half an inch of the tendons of the biceps flexor cruris, the semimembranosus, the semitendinosus, and the gracilis, in a case of such extreme weakness of these muscles, that the tibia and fibula could be so much flexed upwards on the femur as to form a very considerable angle at the knee. Although both legs were similarly affected, I only operated on one, the left, at first, being much the worst. After the operation, having the wounds closed with plaster, and the legs flexed at right angles with the thigh, splints and bandages were then applied to retain it in that position, so as to allow the divided extremities to reunite with the least possible intervention of new tendinous substance between them.

Every thing went on to my entire satisfaction; and in ten days such firm reunion of tendons had taken place as to offer considerable resistance to extending the leg completely. In two days more I did not hesitate to let the patient stand on his legs; when it was most interesting to remark the improvement which had resulted from the operation, this leg being now perfectly straight, the other still falling back in the old form. The day fixed for the operation on the second leg the boy was attacked with scarlatina, which, of course, caused it to be postponed. However, I intend shortly to perform the operation, and have no doubt but the results will be equally satisfactory as those which attended operation on the first leg. I have also operated by incising a portion of tendon of the tibialis anticus, extensor longus pollicis pedis, and of extensor longus digitorum pedis, in cases of paralysis of the muscles on the anterior part of the leg, with similar results.

James Braid, M. R. C. S.

*Case of very early Cadaveric Stiffening.*—Dr. Chowne was induced to submit to the Society a case of extremely early "cadaveric stiffening," because he was not aware of any instance in which it has come on so quickly, and under circumstances so peculiar, and because, moreover, the subject was one of great interest in connection with forensic medicine, perhaps more especially with infanticide. The detail would occupy but a very brief space of time, the facts being few. He received a request in writing from one of the midwives of the Charingcross Hospital, to give his assistance in an obstetric case under her care. Her note stated that the patient had hæmorrhage, and that the child was not born. Upon his arrival at the house in Market-street, Westminster, he found that vigorous natural efforts had terminated the labour, and that the child had been born about three-quarters of an hour. The child was on the midwife's knee; the midwife had put a cap upon it, and was proceeding to put some other article of wearing apparel on. He observed that one arm was a little raised, and the hands partly closed; these circumstances giving it all the appearance of being a living child. He found, however, that it had been still-born, that the birth had taken place three-quarters of an hour before his noticing the state of the arm and hand, and that it had been part of the time in a warm bath. The other limbs were also stiff in about the same degree. He examined the stiffness carefully; it was unequivocal; there was no elasticity, as if the stiffness was the result of spasmodic muscular contraction; a joint being flexed, it remained flexed. The midwife informed him, that she particularly observed the stiffness while the child was in the warm bath. The mother said the child moved very perceptibly to herself not long before its birth. Whether facts tended to weaken or to strengthen existing opinions, they were in either case useful, and must be equally interesting to the profession. This case he submitted to the society, as one which might warrant a degree of circumspection with reference to some general views entertained on the subject of cadaverous rigidity.

Beck says, in speaking of coldness and stiffness, "I may remark that their supervention is far from being uniform; the bodies of the aged take them on much sooner than those of the young, and again the nature of the disease has a manifest influence."

In the work of Paris and Fonblanque, we find it stated as a general rule, that the more sudden the death, the longer is cadaverous stiffness in taking place. This passage is quoted by Beck, without dissent or qualification.

According to Orfila, "if the body of a person suffocated either by a non-respirable gas, or by strangulation, be cold or stiff, we may be certain that more than twelve hours have elapsed since the fatal event; for in death by such causes, the heat of the body is preserved for at least this period." This observation not only tends to fix the date of the event at a certain distance of time from the dissolution, but somewhat more than implies, that there is a *necessary* connection or relation between the departure of warmth and the accession of stiffness.

The remark of Richerand, that "in asphyxia from carbonic acid, the blood preserves its fluidity, the limbs their flexibility, and the body its natural heat, for some hours after death," appears to imply the same relation.

It has been stated, on good authority, that "in all animals, the moment when stiffness commences, is that in which the vital heat begins to be extinct; that the occurrence of cadaveric stiffness is subordinate to the *same causes* as influence the loss of temperature; that old age favours the dissipation of animal *heat;* and, in like manner, it is observed that the muscular rigidity comes on soonest in the bodies of old persons; that the habit of body in which the temperature is longest preserved, is that in which stiffness of the limbs is slowest in its invasion,—thus the bodies of full and fat persons remain longer flexible than those of the meagre and lean; that those diseases which are followed by a longer continuance of warmth, are also remarkable for leaving a corpse in an equal degree flexible; while those in which the body is rapidly cooled, as hæmorrhage for example, favour the approach of the muscular spasm; and after these the body becomes soonest, and sometimes *suddenly*, stiff; that, from this concurrence between the spontaneous cooling of the dead body and the supervention of the cadaveric stiffness, it might be presumed that accidental cooling would be followed by a similar effect, and this is precisely what takes place, for bodies left exposed in cold situations, as on a field of battle, are found to become rapidly stiff.

In distinguishing between the stiffness that occurs in certain forms of syncope and convulsive diseases, it is stated "that it (stiffness from rigidity or convulsion) can never be confounded with cadaveric rigidity, if proper attention be paid to the facts connected with it; for, in the former case, it takes place immediately after the invasion of the disease, and always precedes death, while the body still preserves a considerable *degree of warmth;* whereas the cadaveric stiffness is not observed until some time after death, when the heat of the body is scarcely sensible.

That in all stiffnesses of this kind there is much difficulty in moving a limb; so far it resembles death; but after having bent the limb, on ceasing to apply the force, it immediately flies back to its former position; while the dead limb, on the contrary, remains in the position in which it is placed; and that if death really takes place in any of these convulsive diseases, the muscular contraction ceases with the extinction of the nervous influence to which it was owing, and the true cadaveric stiffness succeeds at the proper time, and pursues its proper course."

It was not his intention to trespass upon the time of the society by any speculation in reference to the real cause or causes of cadaveric stiffening; his object is rather to cite the case as one worthy of attention, both as it regarded the theory of the phenomenon, and as an example of the caution which was always called for in cases of forensic medicine. Had the child in question been born under circumstances suggesting suspicion of infanticide, the unhappy mother would have been placed in a position of danger, and the truth itself would have ministered against her; the cadaveric stiffness would have appeared not to correspond with the time at which the birth actually took place; and the more correctly the time had been stated, the greater would have been the apparent discrepancy: the influence of testimony on the part of the mother, which has the appearance of falsehood under such circumstances, gives increased force to every other unfavourable incident; and the same observation applied to all persons placed in situations of doubt or suspicion.

Although the general tenor of opinions and rules, in relation to cadaveric stiffness, tended to the doctrine that coldness and rigidity were necessarily synchronous, yet some of the facts that had been recorded did not strengthen that conclusion; for example, Professor Louis, from observations made on a large number (more than five hundred) subjects after death, found "that at the moment of the absolute cessation of the vital movements, the articulations began to become stiff, even *before the loss of animal heat.*" Fodré has observed the same, and so far confides not only in the possibility but in the probability of stiffness occurring immediately after death, that he proposes the condition of the limbs, with reference to rigidity, as a test in cases of doubtful death, and observes, that "the flexibility of the limbs is one of the principle signs by which we may judge that a person is not dead, although there is no other sign of life. He (Dr. Chowne) should be unwilling, however, to concur in the faith placed in this sign. Professor Trail states, that he had known rigidity begin before death took place.—(Report of Trial of Robert Reid.)

An interesting case of death from wounds in the neck (inflicted by the deceased himself,) was recorded by Dr. Handyside, in the "Edinburgh Medical and Surgical Journal" (No. 134,) with observations on many points of practical interest. Amongst others, attention is directed to the early period at which stiffness after death took place. "The rigidity of the muscles was complete in this case one hour and a half after death; and the 'vital heat' was evident to the senses after the commencement of the rigidity."

It appears to be extremely probable that cadaveric rigidity of the body occurred *immediately* after death, more frequently than might be generally believed, and that it might do so independently of any necessarry connection with the vital heat. The case which he had submitted to the society appeared to strengthen the opinion, and those which he had adverted to corroborated it.—*Ibid.*

